# Corrigendum: The Vacc-SeqQC project: Benchmarking RNA-Seq for clinical vaccine studies

**DOI:** 10.3389/fimmu.2023.1163550

**Published:** 2023-02-23

**Authors:** Johannes B. Goll, Steven E. Bosinger, Travis L. Jensen, Hasse Walum, Tyler Grimes, Gregory K. Tharp, Muktha S. Natrajan, Azra Blazevic, Richard D. Head, Casey E. Gelber, Kristen J. Steenbergen, Nirav B. Patel, Patrick Sanz, Nadine G. Rouphael, Evan J. Anderson, Mark J. Mulligan, Daniel F. Hoft

**Affiliations:** ^1^ Department of Biomedical Data Science and Bioinformatics, The Emmes Company, LLC, Rockville, MD, United States; ^2^ Division of Microbiology & Immunology, Emory National Primate Research Center, Emory University, Atlanta, GA, United States; ^3^ Department of Pathology & Laboratory Medicine, School of Medicine, Emory University, Atlanta, GA, United States; ^4^ Emory NPRC Genomics Core, Emory National Primate Research Center, Emory University, Atlanta, GA, United States; ^5^ Emory Vaccine Center, Emory University School of Medicine, Atlanta, GA, United States; ^6^ Hope Clinic of the Emory Vaccine Center, Emory University, Atlanta, GA, United States; ^7^ Division of Infectious Diseases, Allergy, and Immunology, Department of Internal Medicine, Saint Louis University School of Medicine, St. Louis, MO, United States; ^8^ McDonnell Genome Institute, Washington University, St. Louis, MO, United States; ^9^ Office of Biodefense, Research Resources and Translational Research, National Institute of Allergy and Infectious Diseases, National Institutes of Health, Rockville, MD, United States; ^10^ Department of Medicine, Division of Infectious Diseases, Emory University School of Medicine, Emory University, Atlanta, GA, United States; ^11^ Center for Childhood Infections and Vaccines (CCIV) of Children’s Healthcare of Atlanta and Department of Pediatrics, Emory University School of Medicine, Atlanta, GA, United States; ^12^ New York University Vaccine Center, New York, NY, United States; ^13^ Department of Molecular Microbiology & Immunology, Saint Louis University, St. Louis, MO, United States

**Keywords:** RNA-Seq, statistical power, ERCC, tularemia vaccine (DVC-LVS), gene filtering, sequencing depth, read length, reproducibility

In the published article, there was an error in the **Figure 7** legend as published. The figure legend effect size values were incorrectly displayed as “>1.25, 51.5, 51.75, 52” instead of “>1.25, ≥1.5, ≥1.75, ≥2”. The corrected **Figure 7** and its caption appear below.

**Figure 7:**
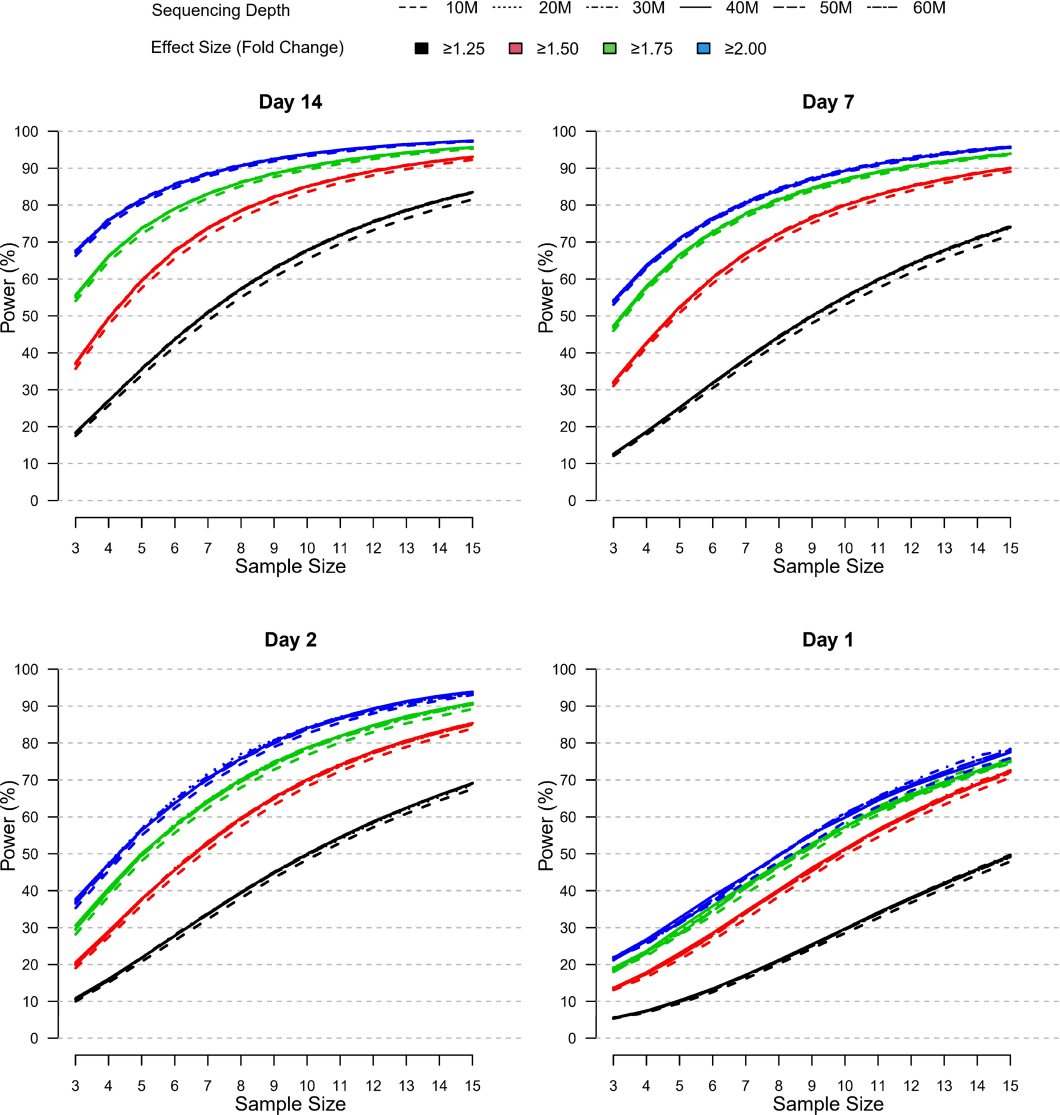
Relative power by sample size, effect size, and sequencing depth at each post-vaccination day as simulated using the modified PROPER R package. Days were sorted by decreasing vaccination effect based on overall fold changes and DEG responses observed for this study (see Figure 3A). Power was assessed for different fold-change cutoffs (indicated by color-coded lines), sequencing depth (as indicated by the line type), and sample size (x-axis).

The authors apologize for this error and state that this does not change the scientific conclusions of the article in any way. The original article has been updated.

