# Supplementary material for: The Vacc-SeqQC project: Benchmarking RNA-Seq for clinical vaccine studies
Source: Front Immunol. 2023 Jan 19;13:1093242. doi: 10.3389/fimmu.2022.1093242 (PMC9893923; doi:10.3389/fimmu.2022.1093242)
Supplement: Supplementary file 1 [file DataSheet_1.docx]

Supplementary Material

# Supplementary Figures and Tables

## Supplementary Figures

**Supplemental Figure 1: Inter-site agreement of RNA-Seq log_2_ counts per million and fold-change estimates improves after filtering out lowly-expressed genes.** LCPM: log_2_ counts per million. LFC: log_2_ fold change. **(A)** Violin plot depicting the distribution of adjusted Euclidean distance between Site 1 and Site 2 of the LCPM for each gene. A total of 49 paired observations were available for each gene. Red dots mark the mean of the distribution, and black diamonds mark the median. Each column shows the distribution after filtering genes at the CPM thresholds indicated on the X axis. **(B)** The same analysis and plotting design as described in (A), but of Pearson correlation between Site 1 and Site 2 of the LCPM for each gene. **(C)** and **(D)** show the same results but calculated on the LFCs. A total of 36 paired observations of LFC values were available for each gene.


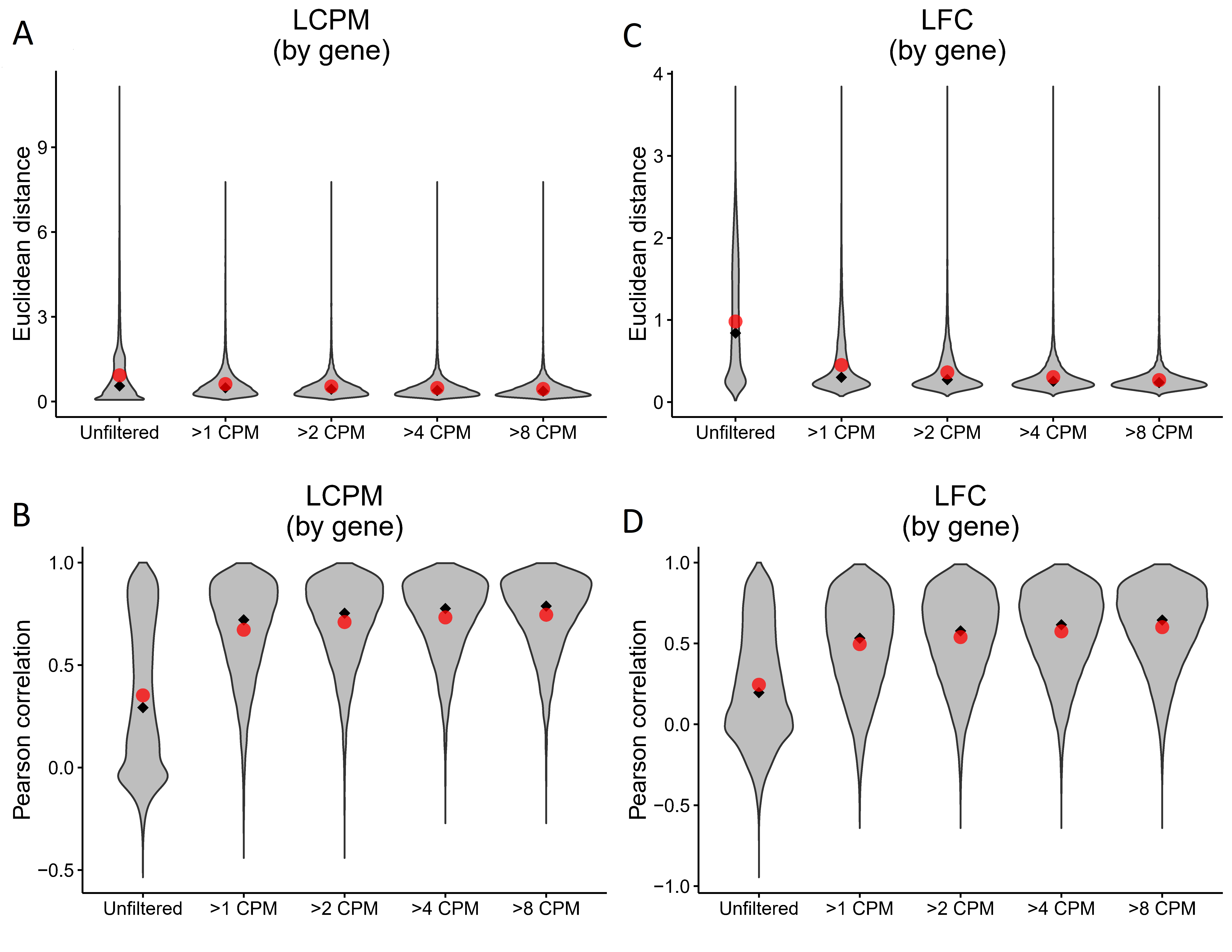


**Supplemental Figure 2: Impact of read length and coverage on DEG detection.** Impact of read length and coverage on the number of DEGs (Site 1). Values for the original dataset, with a median coverage of 31.8 M and a read length of 151 nt, for comparison.

**
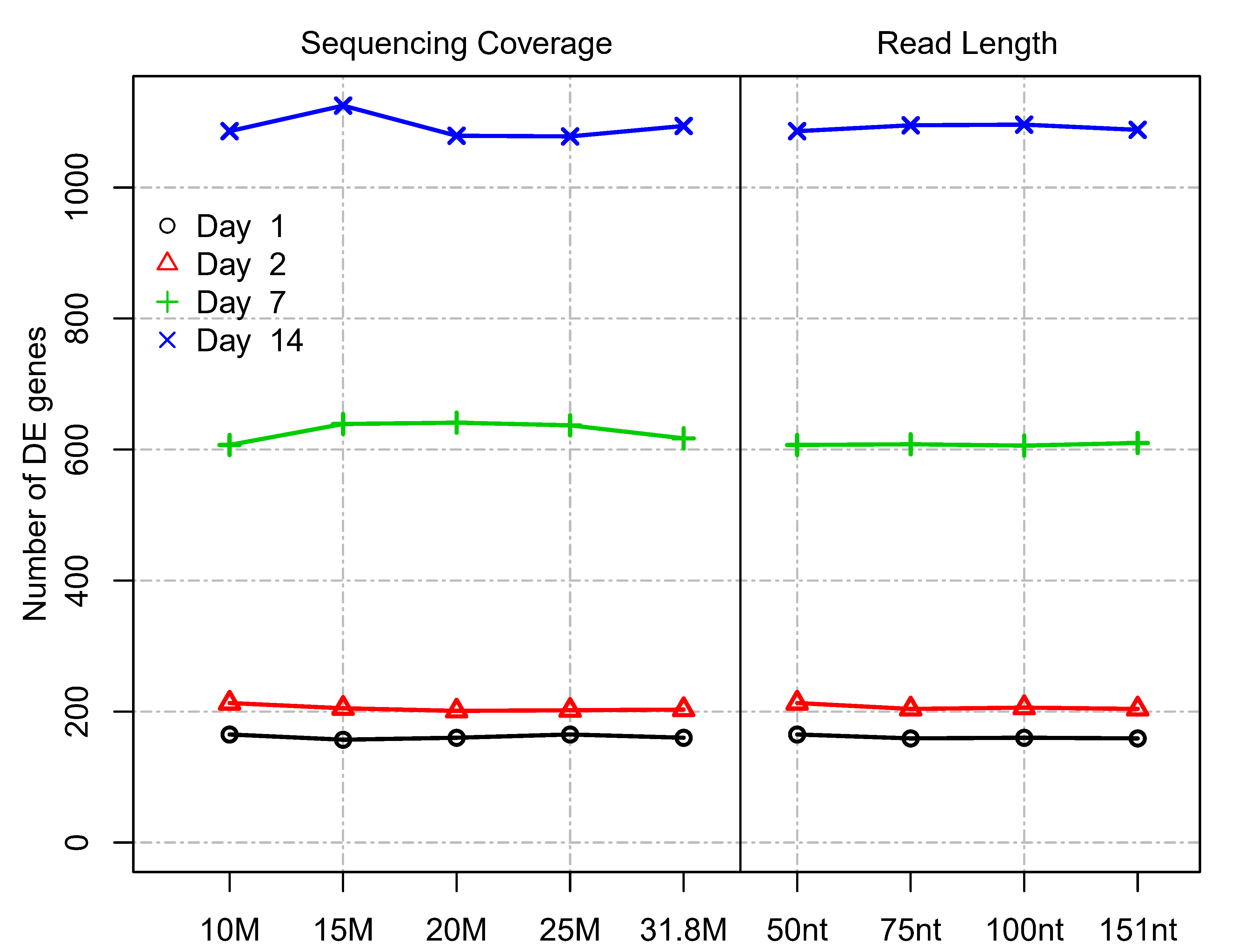
**

**Supplemental Figure 3: Relative false discovery rate by samples size, effect size, and coverage at each post-vaccination day as simulated using the modified PROPER R package.** Days were sorted by decreasing vaccination effect based on overall fold changes and DEG responses observed for this study (see **Figure 3A**). False discovery rate was assessed for different fold-change cutoffs (indicated by color-coded lines), coverage (as indicated by the line type), and sample size (x-axis).


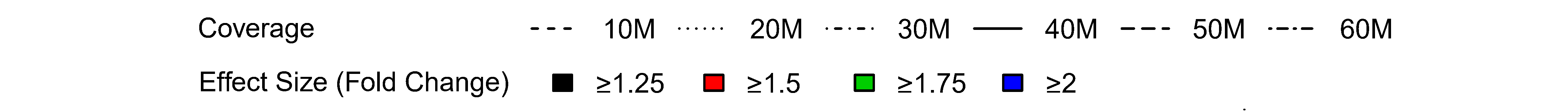

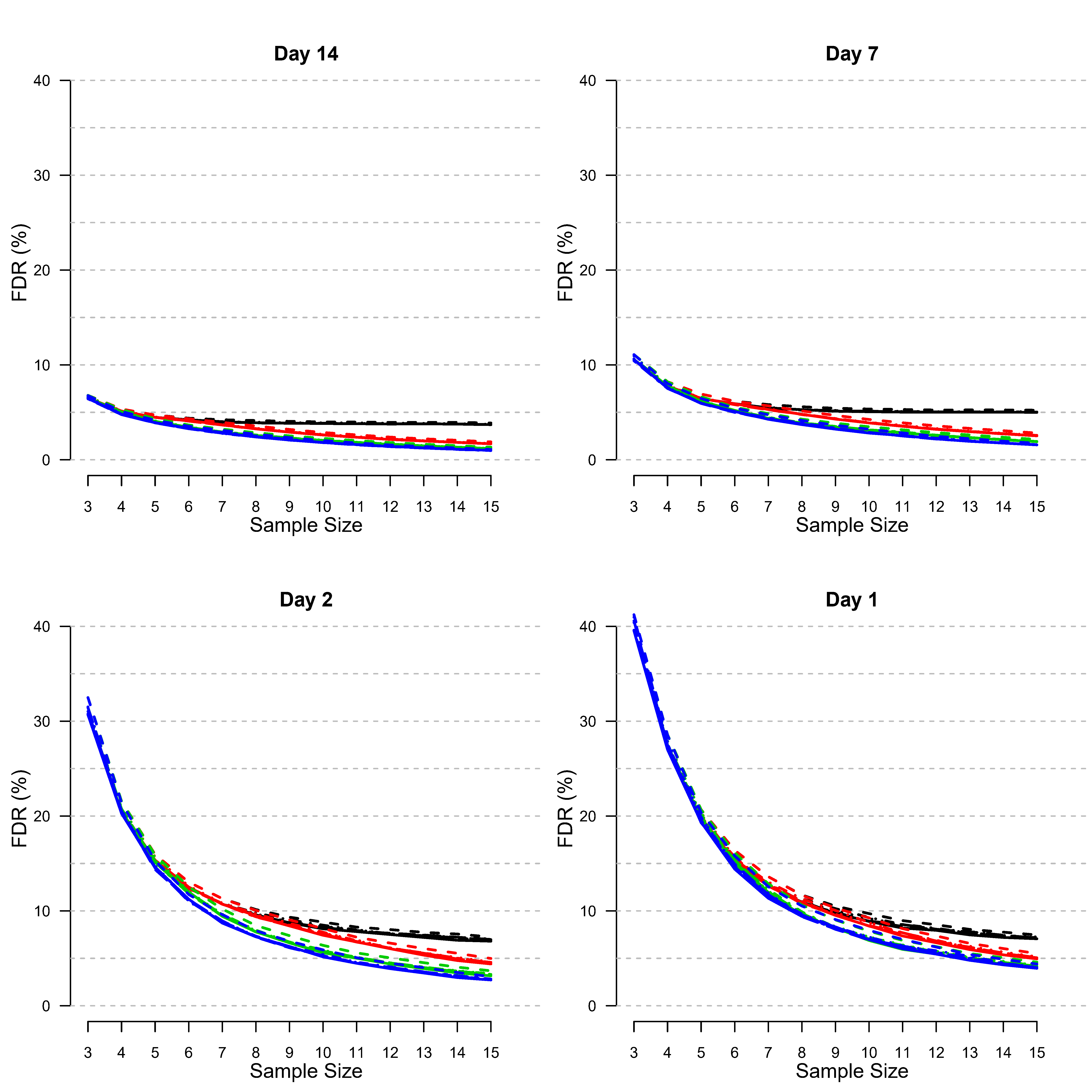


**Supplemental Figure 4: Relative type-I-error rate by samples size, effect size, and coverage at each post-vaccination day as simulated using the modified PROPER R package.** Days were sorted by decreasing vaccination effect based on overall fold changes and DEG responses observed for this study (see **Figure 3A**). Type-I-error rate was assessed for different fold-change cutoffs (indicated by color-coded lines), coverage (as indicated by the line type), and sample size (x-axis).


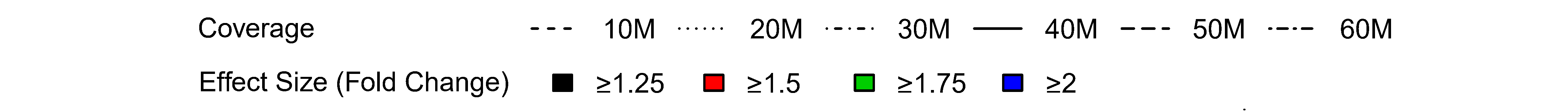


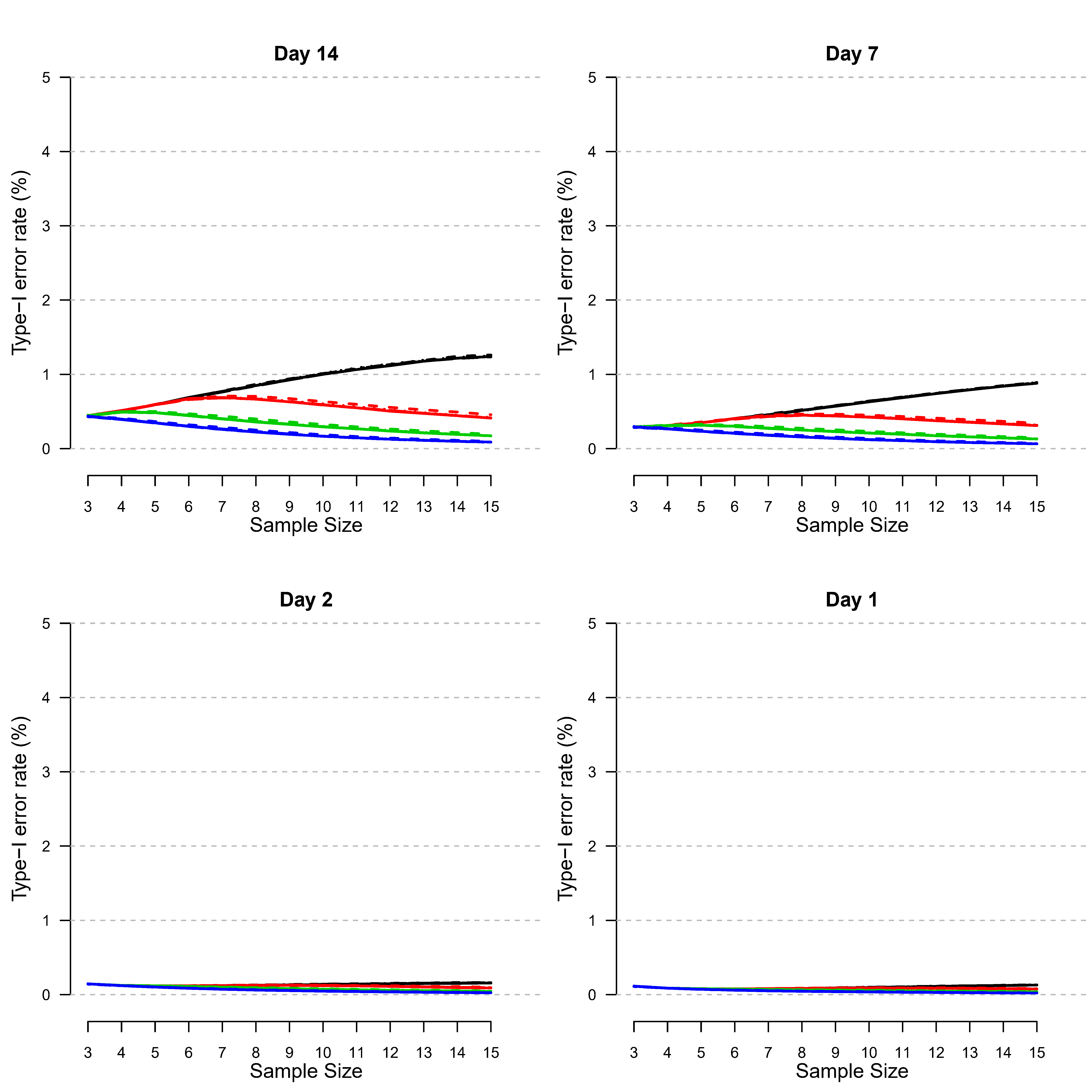


**Supplemental Figure 5: Mean-dispersion trend for each simulated coverage dataset (left) and scatterplot of mean log­_2_ CPM of genes in original dataset compared to each simulated coverage dataset (right).** For each gene in the simulated coverage datasets, the estimated log_2_ mean expression and log_2_ over-dispersion values were calculated; from these points, a loess curve is fit show the mean-dispersion trend at each simulated coverage level (A). The average TMM-normalized log_2_ CPM values are also calculated for each gene across baseline samples in each simulated dataset and compared to the corresponding values from the original dataset (B).


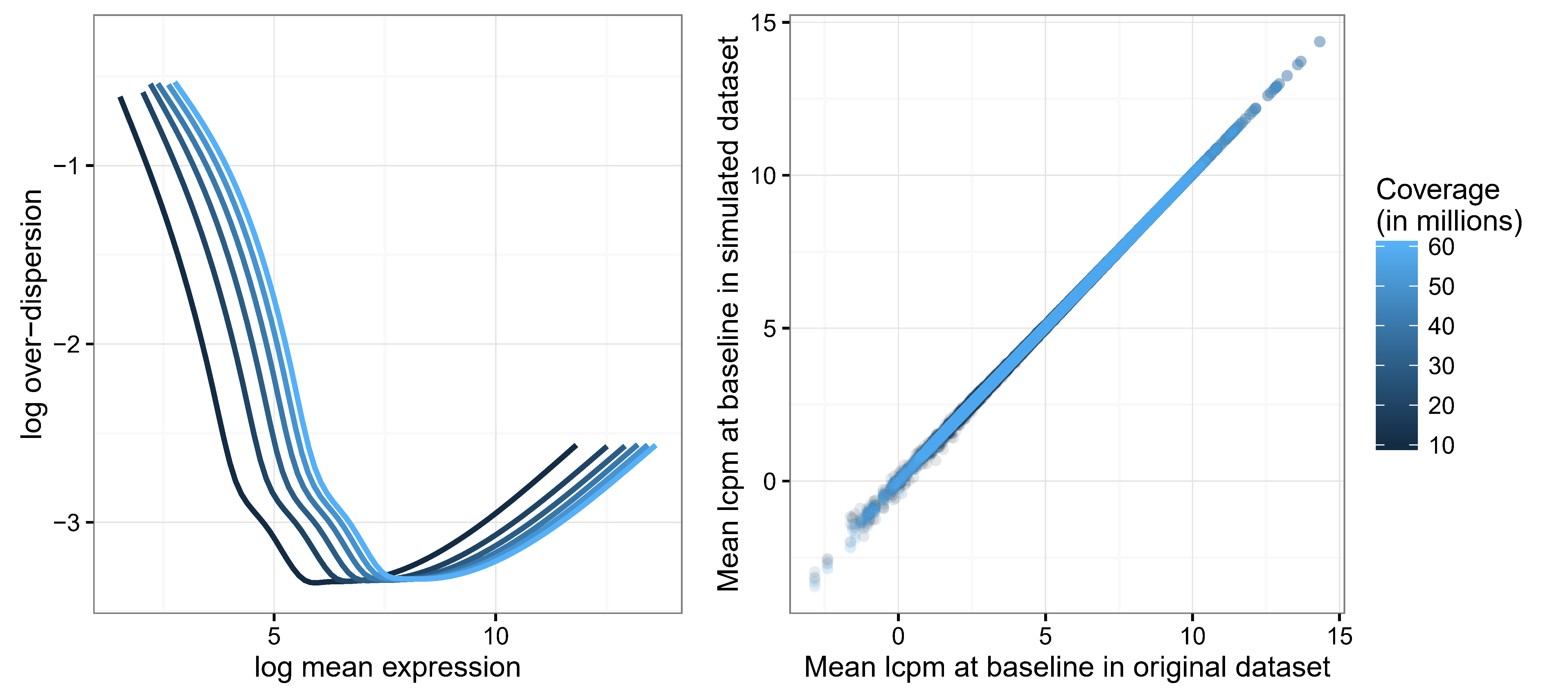


**A B**

**Supplemental Figure 6: Principal component analysis of log_2_ counts per million across sites before and after normalization within and between sites.**

**
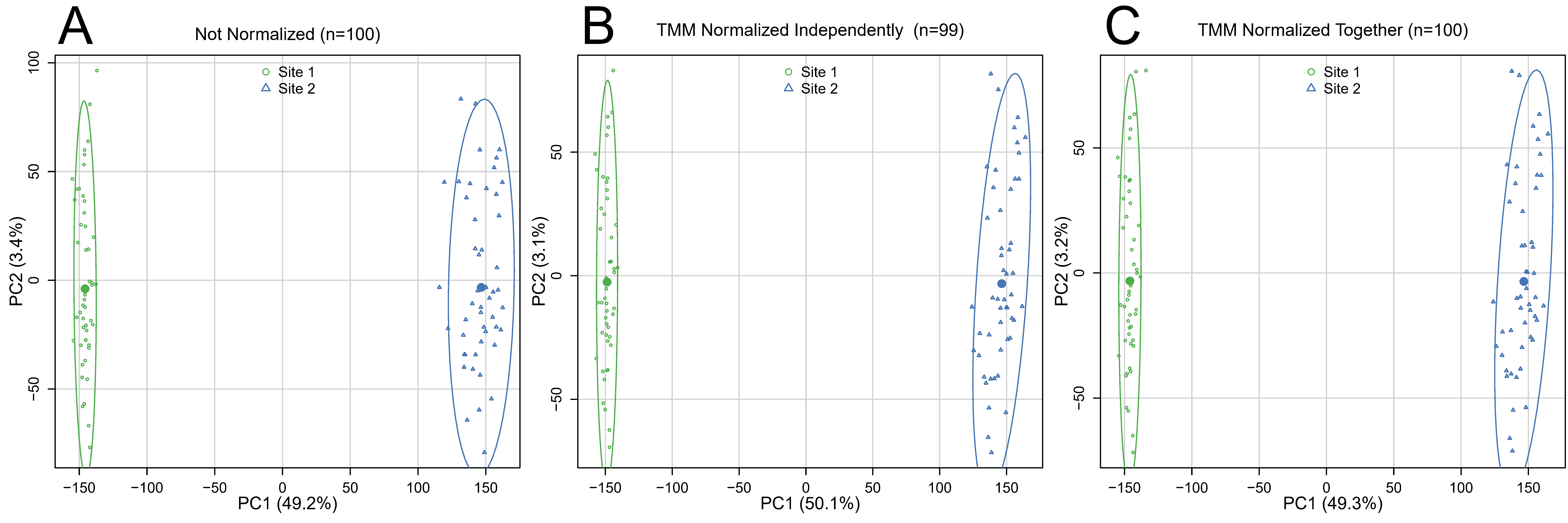
**

**Supplemental Figure 7: Heatmap summarizing fold change responses of the union of DEGs by subject and Site.** Subjects and genes were clustered using Uncentered Pearson Correlation distance in combination with complete linkage clustering.

**
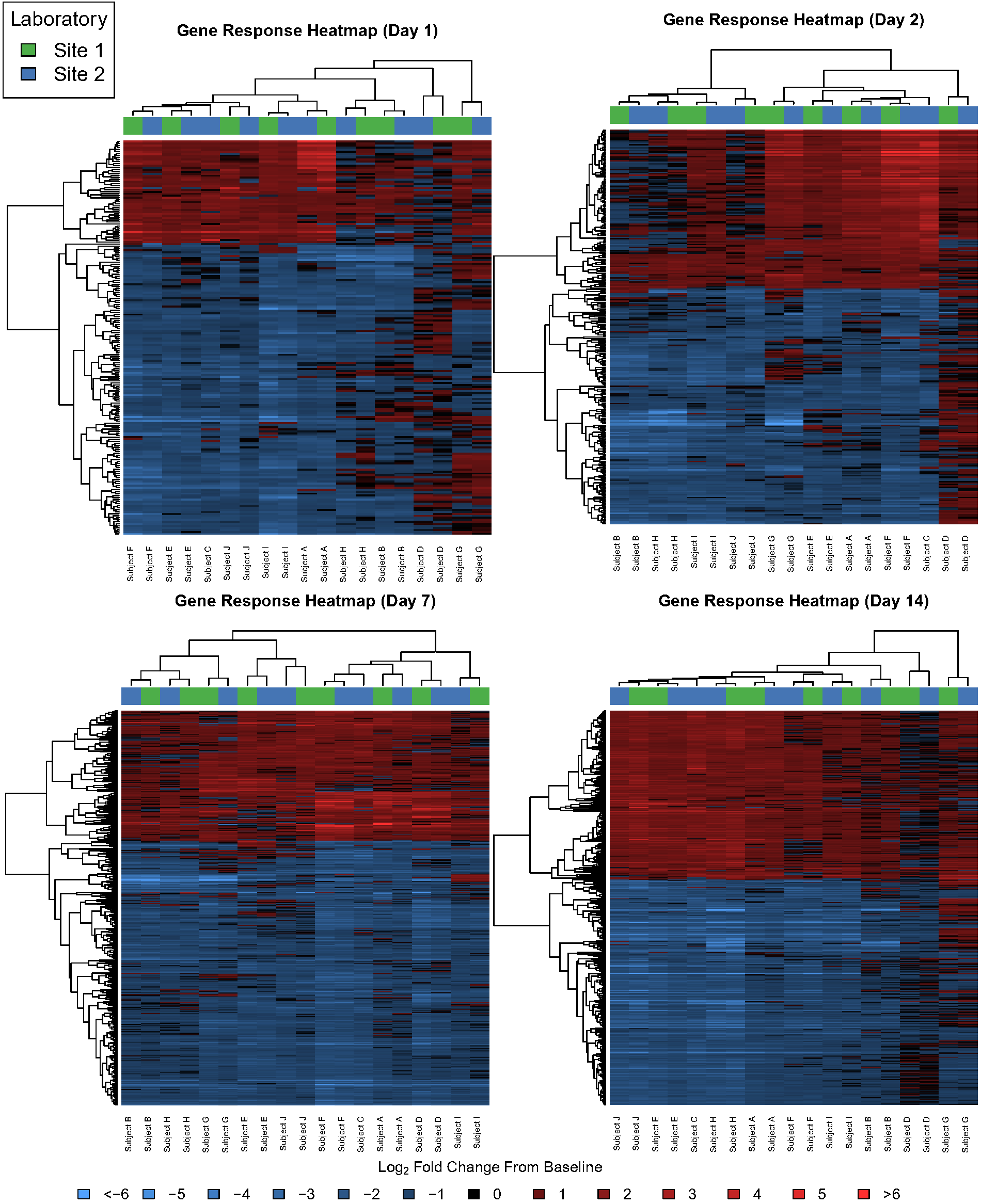
**

## Supplementary Tables

**Supplemental Table 1:** Impact of fold change and FDR-adjusted p-value cutoff on DEG agreement. Total represents reference DEGs. All other columns to the right refer to the comparison group. FC = fold change $\geq$1.5; FDR = FDR-adjusted p-value <0.05.

| **Reference Laboratory** | **Comparison Laboratory** | **Day** | **DE Total** | **Missing**  **(% Total)** | **DE (% Total)** | **Not DE**  **(% Total)** | **Fail FC and FDR**  **(% Not DE)** | **Pass FC and Fail FDR**  **(% Not DE)** | **Fail FC and Pass FDR**  **(% Not DE)** |
| --- | --- | --- | --- | --- | --- | --- | --- | --- | --- |
| Site 1 | Site 2 | Day 1 | 165 | 8 (4.8%) | 90 (54.5%) | 67 (40.6%) | 24 (35.8%) | 2 (3%) | 41 (61.2%) |
| Site 1 | Site 2 | Day 2 | 213 | 10 (4.7%) | 145 (68.1%) | 58 (27.2%) | 23 (39.7%) | 1 (1.7%) | 34 (58.6%) |
| Site 1 | Site 2 | Day 7 | 607 | 41 (6.8%) | 407 (67.1%) | 159 (26.2%) | 20 (12.6%) | 2 (1.3%) | 137 (86.2%) |
| Site 1 | Site 2 | Day 14 | 1086 | 72 (6.6%) | 794 (73.1%) | 220 (20.3%) | 14 (6.4%) | 7 (3.2%) | 199 (90.5%) |
| Site 2 | Site 1 | Day 1 | 155 | 26 (16.8%) | 90 (58.1%) | 39 (25.2%) | 14 (35.9%) | 9 (23.1%) | 16 (41%) |
| Site 2 | Site 1 | Day 2 | 240 | 25 (10.4%) | 145 (60.4%) | 70 (29.2%) | 32 (45.7%) | 6 (8.6%) | 32 (45.7%) |
| Site 2 | Site 1 | Day 7 | 576 | 68 (11.8%) | 407 (70.7%) | 101 (17.5%) | 21 (20.8%) | 6 (5.9%) | 74 (73.3%) |
| Site 2 | Site 1 | Day 14 | 1087 | 119 (10.9%) | 794 (73%) | 174 (16%) | 23 (13.2%) | 4 (2.3%) | 147 (84.5%) |
